# Spontaneous aging of mature amyloids alters structural stability, cytotoxicity, and susceptibility to biological clearance

**DOI:** 10.1038/s41420-026-03245-1

**Published:** 2026-07-07

**Authors:** Maksim I. Sulatsky, Olga V. Stepanenko, Arina A. Kayda, Olesya V. Stepanenko, Ekaterina V. Mikhailova, Anna I. Sulatskaya

**Affiliations:** 1 Laboratory of cell morphology, Institute of Cytology Russian Academy of Sciences, St. Petersburg, Russia; 2 Laboratory of structural dynamics, stability and folding of proteins, Institute of Cytology Russian Academy of Sciences, St. Petersburg, Russia

**Keywords:** Cell death, Protein aggregation, Protein aggregation, Proteolysis

## Abstract

Accumulation of amyloid fibrils is a key factor in the pathogenesis of progressive diseases, including neurodegenerative disorders such as Alzheimer’s and Parkinson’s disease, as well as systemic amyloidosis. Although amyloid deposits are known to persist in vivo for years or even decades, it remains unclear how the structure and biological properties of mature fibrils evolve during prolonged post-assembly residence. Here, we addressed this question using a simplified cell-free aqueous model designed to isolate the intrinsic time-dependent behavior of mature amyloid aggregates from the complexity of the biological milieu. Specifically, we investigated the long-term evolution of two polymorphs of lysozyme amyloid fibrils as model systems with distinct clustering propensities that mimic the diversity of amyloid deposits. We challenge the assumption of amyloid stability by demonstrating their spontaneous degradation over 16 months at physiological temperature, a process we define as amyloid “aging”. This process is characterized by aggregate declustering, fibril shortening, and progressive depolymerization into monomeric subunits, accompanied by a pronounced reduction in intrinsic toxicity across multiple human cell lines. Notably, “aged” fibrils were disassembled and degraded more efficiently than freshly prepared aggregates by the molecular chaperone α-B-crystallin and immune-associated proteases, including matrix metalloproteinase-9 and cathepsins B and D. However, this enhanced degradation revealed a paradox: accelerated processing of “aged” amyloids did not always reduce cytotoxicity and, in some cases, even exacerbated it, consistent with the generation of biologically active fibril-derived species rather than their complete conversion into non-toxic monomers. We provide the first systematic evidence that mature amyloids are dynamic structures undergoing spontaneous degradation that fundamentally alters their cytotoxicity and susceptibility to biological clearance. Our results introduce amyloid aging as a previously underappreciated dimension of amyloid biology and emphasize the need to account for fibril “age” when evaluating pathogenic potential and developing anti-amyloid therapeutic strategies.

**Figure:**
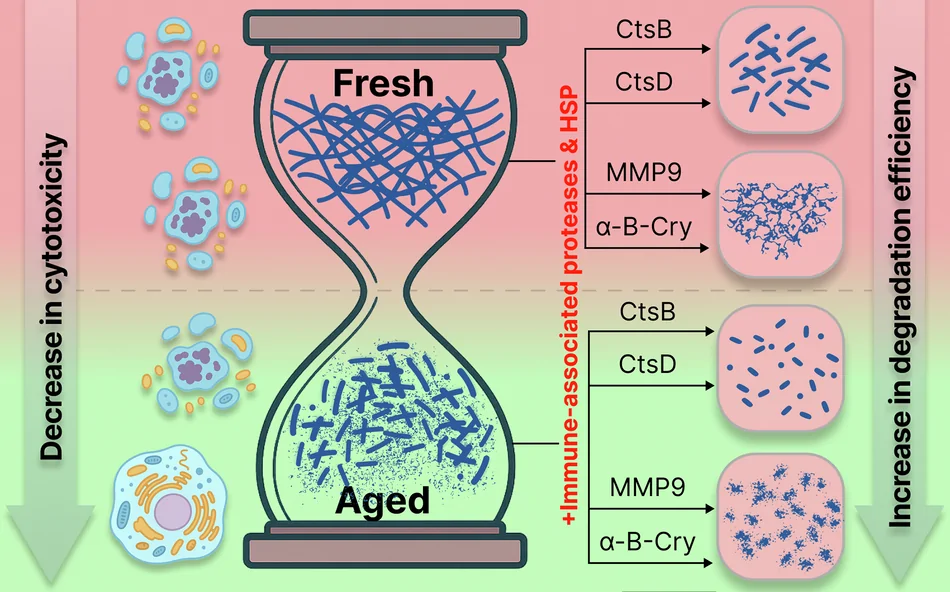
Spontaneous “aging” of amyloid fibrils leads to suprastructural remodeling: declustering, fibril shortening, and attenuated cytotoxicity. While “aged” aggregates are degraded more efficiently by immune-associated proteases and α-B-crystallin than “fresh” fibrils, this enhanced degradation does not reduce cytotoxicity and, in some cases, exacerbates it, highlighting the complex interplay between aggregate maturity and biological outcomes.

Spontaneous “aging” of amyloid fibrils leads to suprastructural remodeling: declustering, fibril shortening, and attenuated cytotoxicity. While “aged” aggregates are degraded more efficiently by immune-associated proteases and α-B-crystallin than “fresh” fibrils, this enhanced degradation does not reduce cytotoxicity and, in some cases, exacerbates it, highlighting the complex interplay between aggregate maturity and biological outcomes.

## Introduction

The accumulation of amyloid fibrils is associated with numerous diseases, including neurodegenerative disorders (Alzheimer’s disease, Parkinson’s disease), systemic amyloidoses, diabetes, and other severe conditions [1–3]. These predominantly incurable diseases arise from the conversion of soluble monomeric proteins into insoluble fibrillar aggregates that accumulate within and outside cells, damaging tissues and organs [4]. Amyloid assemblies exert cytotoxic effects through multiple mechanisms. Soluble oligomeric species are often regarded as the most potent membrane-disrupting forms in many amyloid systems [5–7], particularly with respect to pore formation and ionic imbalance. However, mature fibrils may also contribute to cytotoxicity both directly, through their own interactions with cellular membranes and broader downstream pathogenic effects, and indirectly, by acting as reservoirs or sources of smaller membrane-active species released during remodeling, fragmentation, or partial disassembly, or by catalyzing their generation on fibril surfaces [8–11].

Beyond their multifaceted cytotoxicity [5–8], amyloid fibrils possess a unique property dictated by their cross-β architecture. This structure comprises stacked β-strands oriented perpendicular to the fibril axis and stabilized through steric zipper interactions [12–14]. Such organization confers exceptional mechanical stiffness and thermodynamic stability, comparable to silk or steel on the nanoscale [15–17]. While the kinetics of amyloid formation are well-characterized, the post-assembly fate of these aggregates remains an overlooked dimension of amyloid biology.

Clinical and experimental evidence confirms that the high stability of amyloid fibrils enables them to persist in biological environments and complicates their degradation and clearance in disease contexts [18, 19]. For example, in AL amyloidosis, rapid disease progression limits median patient survival to 1–2 years, defining the corresponding persistence period of amyloid aggregates, predominantly in the heart, kidneys, liver, spleen, and gastrointestinal tract [20, 21]. Conversely, in transthyretin (ATTR) amyloidosis, stable deposits persist in the heart and other organs for 5–15 years post-diagnosis [22]. Finally, amyloid-β fibril accumulation in the brain begins 15–20 years before clinical manifestation of Alzheimer’s disease and continues throughout disease progression [23, 24].

While mature amyloid plaques were viewed as static end-products of protein aggregation, emerging structural evidence challenges this paradigm, demonstrating that amyloid assemblies remain dynamic systems capable of conformational remodeling and polymorph switching even after deposition [25, 26]. Despite this paradigm shift, experimental data regarding the specific functional consequences of such maturation remain scarce. Determining whether these long-lived supramolecular protein assemblies retain the integrity and cytotoxic properties of their freshly formed counterparts is essential for predicting the biological fate of persistent deposits. Furthermore, the structural state of amyloid fibrils dictates their susceptibility to clearance mechanisms. Therapeutic strategies, including immunotherapies and enzymatic augmentation, aim to disassemble these persistent deposits [27–29]. However, if prolonged persistence alters fibril stability or clustering, it may fundamentally change the outcome of such interventions. A key safety concern is whether the partial degradation of remodeled structures detoxifies the tissue or, conversely, releases active membrane-disrupting amyloid species [8, 19]. Understanding the interplay between fibril persistence duration and proteolytic susceptibility is therefore crucial for the design of safe clearance strategies.

To address these questions, we established a long-term in vitro model using lysozyme amyloid fibrils accumulated in systemic lysozyme amyloidosis [30, 31]. Lysozyme serves as a robust system for studying amyloid persistence, allowing the generation of structurally distinct polymorphs with controlled clustering degrees that recapitulate the diversity of amyloid deposits observed in vivo [32–35]. By monitoring these structures over a 16-month incubation in a defined aqueous cell-free environment at physiological temperature, we characterized the spontaneous time-dependent suprastructural transition of amyloid assemblies and evaluated how this process modulates their cytotoxicity and interaction with key cellular degradation factors, including lysosomal cathepsins, matrix metalloproteinase-9 (MMP-9), and the chaperone-like protein α-B-crystallin. These results enabled us to analyze key features of amyloid aggregate “aging”, providing insight into understanding amyloidosis pathogenesis and progression, developing effective therapeutic strategies targeting amyloid fibrils of varying “maturity”, and establishing relevant protocols for testing the efficacy of novel amyloid-degrading compounds.

## Results

### Structural and morphological changes in mature amyloid fibrils during prolonged incubation

**Fig. 1:**
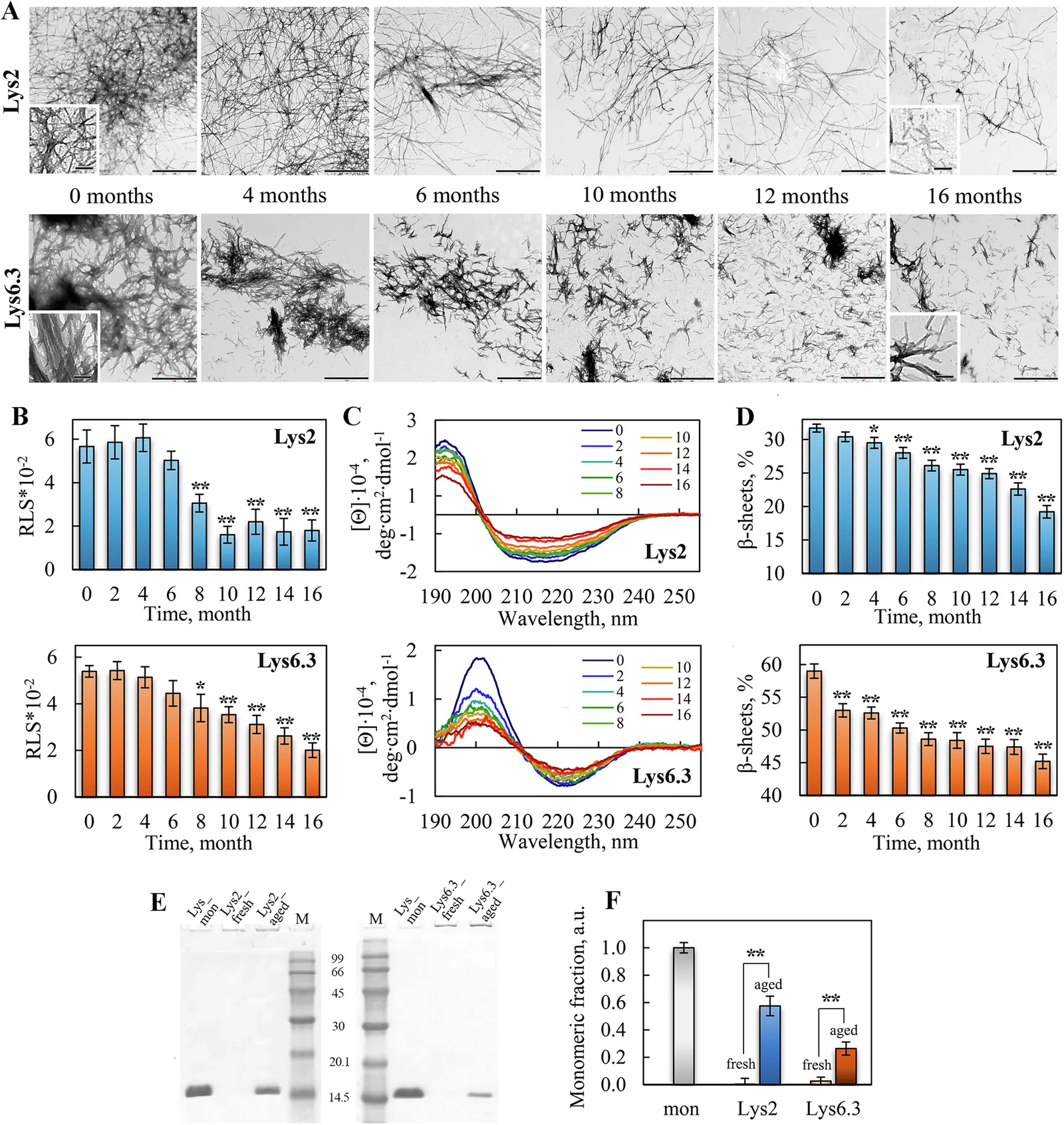
Changes in morphology, size, and structure of lysozyme amyloid fibrils incubated at physiological temperature. **A** Visualization by transmission electron microscopy. Microphotographs of amyloids prepared at acidic pH (Lys2) and at near-neutral pH (Lys6.3) fibrils are shown in the top and bottom rows, respectively. The incubation times are indicated between the rows. Scale bars are equal to 1 µm. Insets show images of representative samples at higher magnification. Scale bars are equal to 0.1 µm. **B** Rayleigh light scattering (RLS), **C** far-UV CD spectra, and **D** β-sheet content calculated using the BeStSel server. Data for Lys2 and Lys6.3 are presented in the top and bottom rows, respectively. The color designations for **C** are indicated near the spectra. **B**, **D** Data are presented as mean ± SD, *n* = 5 at each time point. Statistical comparisons were performed separately for Lys2 and Lys6.3 fibrils using Brown–Forsythe and Welch ANOVA tests, followed by Dunnett’s T3 multiple comparisons test with each time point compared to the corresponding 0-month sample. Significance levels are indicated as follows: (**) *p* < 0.01, (*) *p* < 0.05. For clarity, non-significant results are not shown. **E** Pseudo-native 17% SDS-PAGE of the freshly prepared (“fresh”) and long-incubated (“aged”) lysozyme amyloid fibrils. Molecular weights of the marker proteins (M) are indicated between the two gels. Samples of “fresh” and “aged” Lys2 (Lys2_fresh, Lys2_aged) and Lys6.3 (Lys6.3_fresh, Lys6.3_aged) amyloids were loaded in lanes 2 and 3 of the left gel and lanes 3 and 4 of the right gel. As a control, monomeric protein (Lys_mon) was loaded at a concentration equal to that of the amyloids. **F** Quantification of protein monomer content in the samples of “fresh” and “aged” Lys2 (Lys2_fresh, Lys2_aged) and Lys6.3 (Lys6.3_fresh, Lys6.3_aged) amyloids estimated by ImageJ. The intensity of the lysozyme monomer bands in amyloid samples at different time points was normalized to the band intensity of the native monomeric protein (mon). Data are presented as mean ± SD (*n* = 5). Statistical comparisons between fresh and aged samples were performed using Welch’s t-test. Significance levels are indicated as follows: (**) *p* < 0.01.

We studied two lysozyme amyloid fibril polymorphs distinguished by structural differences that determine their clustering propensity. Freshly prepared mature lysozyme amyloids formed at pH 2.0 and transferred to Milli-Q water after fibrillogenesis (Lys2) consisted predominantly of individual fibrils, some assembled into small clusters (Fig. 1A, 0 months, top row). In contrast, lysozyme fibrils formed at pH 6.3 and transferred to Milli-Q water (Lys6.3) exhibited substantially greater propensity to form large and dense clusters (Fig. 1A, 0 months, bottom row). After transfer to Milli-Q water, fibrils were incubated at 37 °C for 16 months until observed changes plateaued. Samples were stored in sealed vials in darkness with pH monitoring throughout the experiment. During this period, no detectable pH drift, gross sample-volume loss, or increase in protein absorbance at 280 nm was observed. End-point sterility testing of representative fresh and 16-month-aged Lys2 and Lys6.3 samples on LB agar revealed no colony growth during 72 h at 37 °C (Fig. S1). We analyzed fibril size, structure, and properties during incubation using spectroscopic, microscopic, electrophoretic, and colorimetric techniques.

Transmission electron microscopy (TEM) revealed distinct morphological changes after 4 months of incubation. The Lys2 samples exhibited fibril declustering (Fig. 1A, 4 months, top row), while Lys6.3 samples showed reduced cluster size and the appearance of small fibrillar fragments (Fig. 1A, 4 months, bottom row). After 6 months, both samples exhibited shortened fibrils. As incubation progressed, the proportion of shortened fibrils increased markedly, accompanied by a further decrease in their size. TEM data correlated with substantial decreases in Rayleigh light scattering (RLS) during prolonged incubation (Fig. 1B). RLS reduction in both samples began at ~6 months.

We recorded far-UV circular dichroism (CD) spectra to obtain semiquantitative information on changes in the secondary-structure signatures of the fibril samples. Because far-UV CD analysis of fibrillar material is more challenging than that of soluble proteins due to possible light-scattering artifacts, spectra were recorded at relatively low protein concentrations (0.15–0.2 mg/mL), and only spectra showing near-zero ellipticity in the 250–260 nm region after baseline correction were used for further analysis. The CD data were interpreted together with TEM, ThT fluorescence, and electrophoretic results. Fresh aggregates of both polymorphs displayed a pronounced minimum ~220 nm, consistent with β-structure-rich amyloid assemblies (Fig. 1C). During incubation, the amplitude of this signal decreased, indicating a partial loss of ordered β-structure contribution in the samples. BeStSel analysis suggested that these spectral changes followed different apparent trajectories in the two polymorphs: Lys2 showed a relative increase in α-helical-like/non-β contributions, whereas Lys6.3 showed a stronger increase in β-turns and in the BeStSel “Others” component, which includes 3₁₀-helices, π-helices, β-bridges, bends, loop/irregular, and structurally unresolved regions (Figs. 1D and S2).

Because TEM revealed no conversion of amyloids into amorphous aggregates, whereas CD spectroscopy indicated a loss of β-sheet structure, we hypothesized that fibrils depolymerize into monomeric or oligomeric subunits. To test this hypothesis and quantify depolymerization efficiency, we analyzed pseudo-native electrophoresis results (Fig. 1E) using ImageJ (Fig. 1F). Monomeric protein content increased substantially after 16 months of incubation: ~24% for Lys6.3 and 57% for Lys2.

**Fig. 2:**
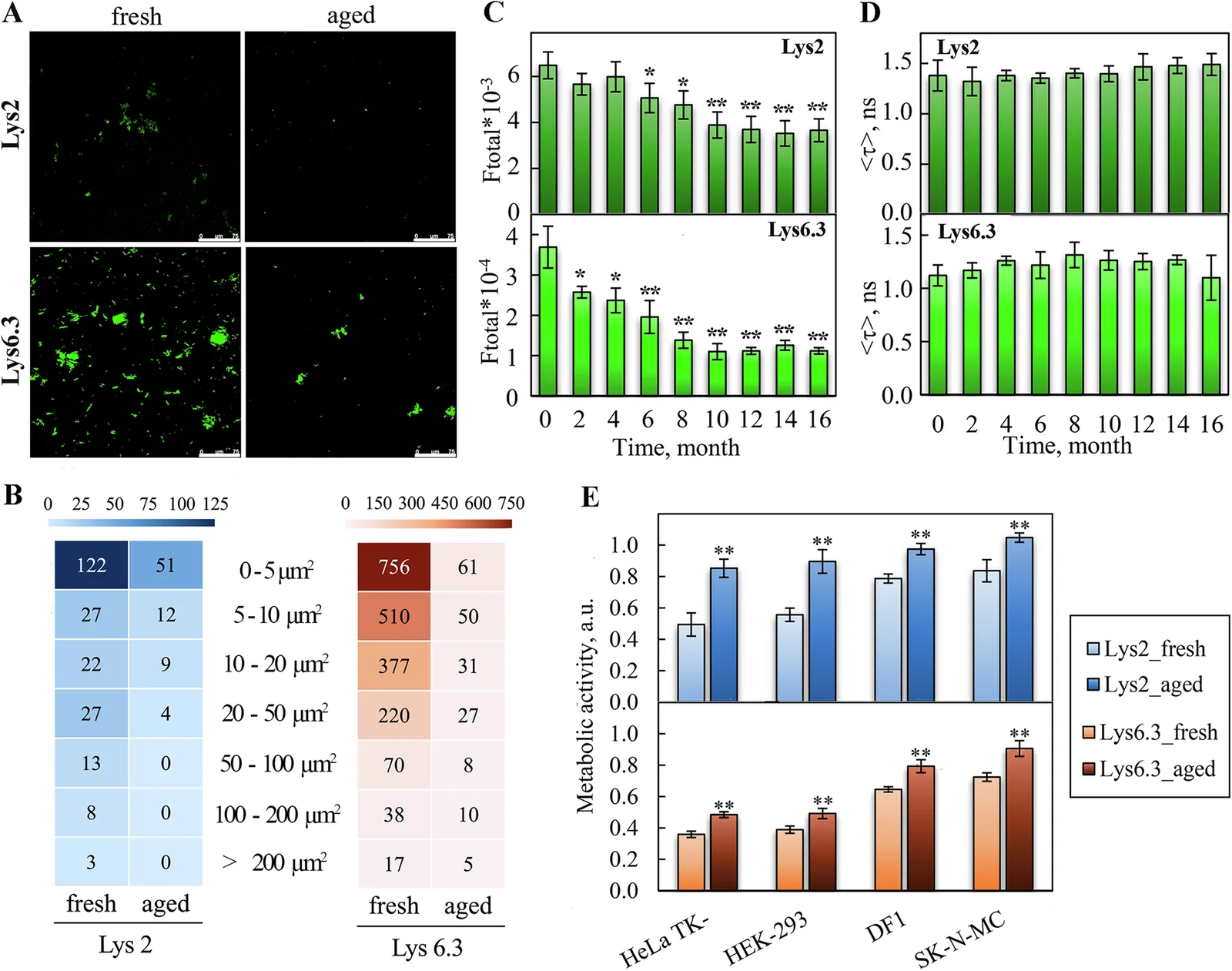
Changes in properties of lysozyme amyloid fibrils incubated at physiological temperature. **A** Binding of fibrils to amyloid-specific probe ThT. Visualization of amyloid aggregates stained with ThT by confocal laser scanning microscopy is presented. Microphotographs of “fresh” and “aged” amyloid aggregates are presented in the left and right column, respectively. Scale bars are equal to 75 µm. **B** Heat maps of the size distribution of ThT-positive amyloid fibril clusters in equal confocal microscopy fields for fresh and aged Lys2 and Lys6.3 samples. The numbers inside the cells indicate the counts of aggregates within the indicated size ranges. **C** Total fluorescence intensity (*F*_total_) and **D** intensity-weighted mean fluorescence lifetime (<*τ*>) of ThT bound to fibrils. **C**, **D** Data are presented as mean ± SD, *n* = 5 at each time point. Statistical comparisons were performed separately for Lys2 and Lys6.3 fibrils using Brown–Forsythe and Welch ANOVA tests, followed by Dunnett’s T3 multiple comparisons test with each time point compared to the corresponding 0-month sample. Significance levels are indicated as follows: (**) *p* < 0.01, (*) *p* < 0.05; ns, not significant. For clarity, non-significant results are not shown. **E** Cytotoxicity of lysozyme fibrils incubated at physiological temperature. Relative metabolic activities of various cell lines–cervical epithelioid carcinoma (HeLa TK-), human embryonic kidney (HEK-293), dermal fibroblast (DF1), and neuroepithelioma (SK-N-MC) cell lines–after 24 h exposure to “fresh” and “aged” amyloids formed from Lys2 (Lys2_fresh, Lys2_aged) and Lys6.3 (Lys6.3_fresh, Lys6.3_aged) are presented. All values are normalized to the metabolic activity of control cells treated with non-fibrillar protein in the same buffer and at the same concentration as the amyloid fibrils. Data are presented as mean ± SD (*n* = 5). Statistical comparisons between fresh and aged samples were performed using Welch’s *t* test. Significance levels are indicated as follows: (**) *p* < 0.01. Lys2 and Lys6.3 data are shown in the top and bottom rows, respectively.

We also examined how the amyloid-specific dye thioflavin T (ThT) interacts with the studied aggregates. This dye binds to channels formed by 4–5 β-strands parallel to the fibril axis [36], resulting in a dramatic increase in fluorescence quantum yield compared to the free dye in aqueous solution (*q* = 0.0001) [37]. When the dye is not only bound to individual fibrils but is additionally confined within a fibrillar cluster, its local microenvironment becomes more rigid, further enhancing fluorescence intensity [38]. ThT thus enables analysis of both individual fibril and cluster degradation. These properties allowed us to visualize ThT-stained amyloid samples by confocal laser scanning microscopy (CLSM) (Fig. 2A). Analysis of the resulting images confirmed fibrillar cluster degradation and progressive reduction in cluster number over time (Fig. 2B). In addition, we were able to analyze the dynamics of fibril degradation using fluorescence spectroscopy. Fluorescence intensity of ThT bound to Lys2 amyloids decreased gradually (Fig. 2C, top panel), consistent with the slow dissociation of individual fibril into monomeric subunits. These changes began at approximately 6 months of incubation. For Lys6.3 amyloids, we observed rapid decline of ThT fluorescence during the first months (Fig. 2C, bottom panel), consistent with TEM data showing cluster size reduction during this period (in which, as noted above, the dye fluoresces most intensely). Subsequent decline was more gradual, confirming predominant fibril dissociation as observed for Lys2 amyloids. After 10 months, ThT fluorescence intensity in Lys6.3 samples plateaued (as in Lys2 samples), suggesting substantial deceleration of fibril monomerization.

ThT fluorescence decay curves enabled assessment of the intensity-weighted mean excited-state lifetime of dye bound to amyloids (Fig. 2D). This parameter remained essentially unchanged over time, indicating that the microenvironment of bound dye was preserved. Thus, the local ThT-binding environment within the residual fibrillar population appears to remain largely preserved despite the observed declustering and partial dissociation. These data agree with TEM results showing that fibril fragments formed during aggregate degradation retain their fibrillar morphology. These observations suggest that the secondary-structure changes detected by CD spectroscopy (Fig. 1D) largely reflect changes in non-fibrillar species formed during fibril dissociation, rather than major alterations in the remaining ThT-binding fibrillar population. Thus, in Lys2, the apparent increase in α-helical-like contribution should therefore not be interpreted as α-helical conversion of the intact fibrillar core. Rather, it is consistent with a stronger contribution of released monomeric and low-order non-fibrillar lysozyme species to the total CD spectrum. Such species need not preserve the β-rich fibrillar conformation and may display more native-like secondary-structure features than the amyloid fibrils themselves [39, 40]. High-resolution cryo-EM analysis could further define whether long-term aging affects fibril-core architecture or selectively remodels suprastructural organization.

### Comparison of the cytotoxic effects of “fresh” and “aged” lysozyme amyloids

We assessed the cellular effects of “fresh” and “aged” (incubated in a defined aqueous cell-free environment at physiological temperature for 16 months) lysozyme amyloid fibrils using the colorimetric MTT assay. To comprehensively evaluate amyloid cytotoxicity, we employed a diverse panel of human cell lines: human embryonic kidney cells (HEK-293), cervical epithelioid carcinoma cells (HeLa TK-), neuroepithelioma cells (SK-N-MC), and dermal fibroblasts (DF1). The SK-N-MC cell line exhibits pronounced neuronal properties and is widely used to study neurotoxicity and mechanisms of amyloid action [41–43]. The HeLa TK- and HEK-293 lines are extensively used to study cell cycle regulation and apoptosis, enabling assessment of amyloid effects on proliferation and survival of non-neuronal cells [44–48]. Finally, the embryonic DF1 line, widely used to study cellular processes and developmental mechanisms, broadens the spectrum of cell models, enhancing the universality of the cytotoxicity assessment for the investigated amyloids and their degradation products [49, 50].

MTT assay data revealed differential effects of “fresh” lysozyme amyloids on cell viability across cell lines of different origins, consistent with published findings [51–53]. Both “fresh” Lys2 and Lys6.3 amyloids exhibited the most pronounced cytotoxicity toward HEK-293 and HeLa TK- lines (Fig. 2E, light blue and light orange bars). Changes in cytotoxicity following prolonged incubation at physiological temperature were consistent across both fibril types: all tested cell lines showed higher viability when exposed to “aged” amyloids (Fig. 2E, dark blue and dark orange bars) compared to “fresh” amyloids. This finding agrees with our conclusion, based on microscopic and spectroscopic data (see section 1.1), that fibrils degrade during incubation at physiological temperature. Lys2 fibrils displayed more substantial cytotoxicity reduction (Fig. 2E), consistent with their more efficient dissociation into non-toxic monomers compared to Lys6.3 amyloids (Fig. 1F).

### Analysis of amyloid fibril degradation at different maturity stages by proteolytic enzymes

After 16 months of incubation, we still observed intact aggregates in samples that appeared resistant to spontaneous degradation upon further incubation under the same conditions (given the marked deceleration of their breakdown). We therefore tested their resistance to external perturbations, specifically to macromolecules present in cells. “Aged” amyloids were exposed to three lysosomal proteases involved in cellular immune responses: the cysteine protease cathepsin B (CTSB), the aspartate protease cathepsin D (CTSD), and MMP-9, as well as the small heat shock protein with chaperone activity, α-B-crystallin (α-B-Cry). Experiments were conducted under optimal conditions for each factor: pH 7.4 for MMP-9, pH 5.5 for CTSB, pH 4.5 for CTSD, and pH 7.4 for α-B-Cry. The stability of amyloid fibrils under these conditions is supported by previous studies using freshly prepared fibrils [51–54] and by TEM analysis of their morphology after resuspension in the corresponding factor-compatible buffer solutions in the absence of CTSB, CTSD, MMP-9, or α-B-crystallin (Fig. S3).

**Fig. 3:**
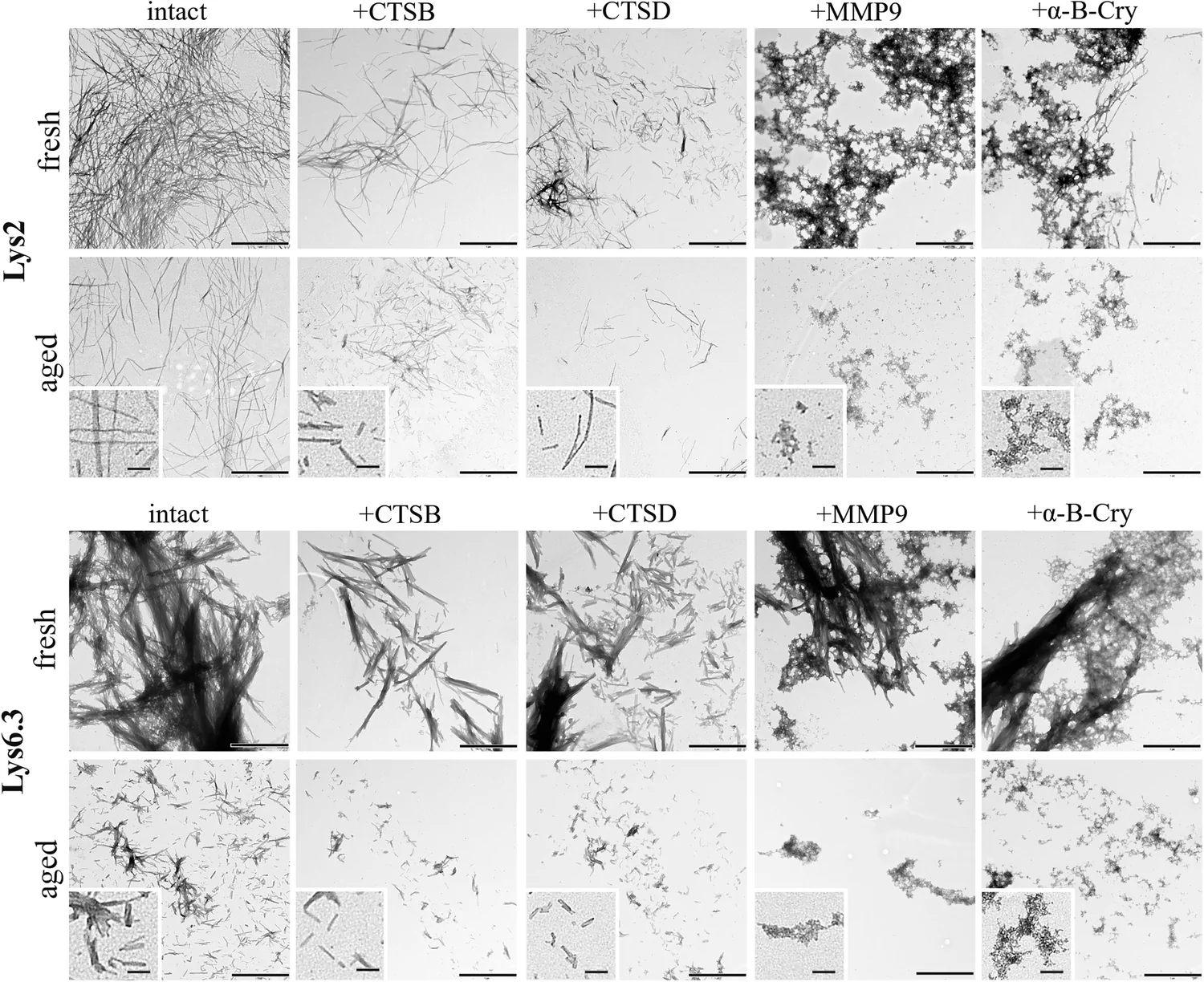
Visualization of “fresh” and “aged” lysozyme amyloid fibrils by transmission electron microscopy before (intact) and after exposure to CTSB, CTSD, MMP-9, and α-B-crystallin (α-B-Cry). Microphotographs of Lys2 and Lys6.3 fibrils are presented in the top and bottom rows, respectively. Scale bars are equal to 1 µm. Insets show images of representative samples at higher magnification. Scale bars are equal to 0.1 µm.

TEM analysis revealed that exposing “fresh” Lys2 and Lys6.3 fibrils to CTSB and CTSD resulted in the shortening of individual fibrils, a reduction in overall fibril abundance, and the declustering of aggregates (Fig. 3, +CTSB/ + CTSD). These observations are consistent with previously published data [51, 53]. Lys6.3 fibril degradation was less efficient than Lys2 degradation. The more extensively clustered Lys6.3 amyloid fibrils likely possess enhanced resistance to proteolytic degradation due to tight protofilament packing and extensive intermolecular interactions that stabilize their structure. Such clustering creates steric barriers that limit protease access to cleavage sites. “Aged” amyloid degradation by CTSB and CTSD followed similar mechanisms as observed for “fresh” amyloids. However, in most cases, “aged” fibril fragments after these treatments were smaller and less clustered than “fresh” fibril degradation products. Thus, the pre-existing partial disruption of “aged” fibrils facilitated more efficient proteolytic degradation compared to “fresh” amyloids.

The degradation mechanism of both lysozyme amyloid types by MMP-9 and α-B-Cry differed from that observed with CTSB and CTSD. We observed efficient “loosening” and structural disorganization of amyloids without substantial reduction in large cluster size (Fig. 3), also consistent with literature data [52, 54]. Moreover, samples containing “fresh” amyloids (Fig. 3) retained substantial amounts of intact (non-degraded) amyloids after MMP-9 and α-B-Cry treatment (particularly pronounced for Lys6.3). Conversely, “aged” Lys2 and Lys6.3 fibrils were degraded more efficiently than the freshly prepared aggregates, mirroring the pattern observed with CTSB and CTSD. We observed complete disruption of their ordered morphology and marked reduction in degradation product size compared to “fresh” amyloids.

Given the significant reduction in fibril density observed by TEM for “aged” Lys2 (Fig. 3), we employed pseudo-native 17% SDS-PAGE to assess potential monomerization or oligomerization (Fig. S4). We found that monomer content in samples after treatment did not increase for any external factor compared to intact “aged” amyloids, and oligomers smaller than 220 kDa did not form.

**Fig. 4:**
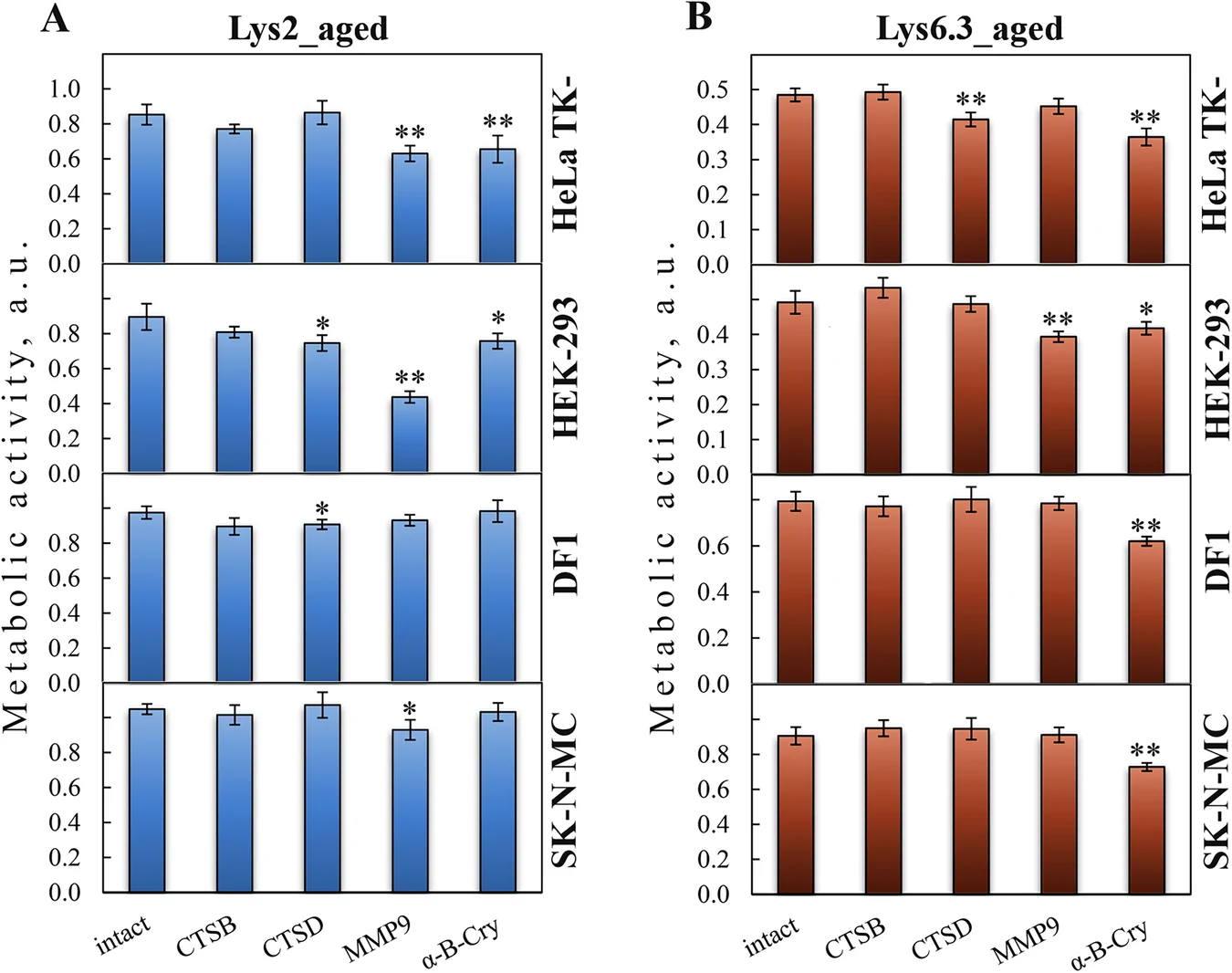
Cytotoxicity of “aged” lysozyme amyloid fibrils before (intact) and after exposure to CTSB, CTSD, MMP-9, and α-B-crystallin (α-B-Cry). Relative metabolic activities of various cell lines–cervical epithelioid carcinoma (HeLa TK-), human embryonic kidney (HEK-293), dermal fibroblast (DF1), and neuroepithelioma (SK-N-MC) cell lines–after 24 h exposure to “aged” amyloids formed from **A** Lys2 and **B** Lys6.3 are presented. All values are normalized to the metabolic activity of control cells treated with non-fibrillar protein in the same buffer and at the same concentration as the amyloid fibrils. Data are presented as mean ± SD, *n* = 5. Statistical comparisons were performed separately for Lys2 and Lys6.3 fibrils using Brown–Forsythe and Welch ANOVA tests, followed by Dunnett’s T3 multiple comparisons test with each treatment compared to the intact sample. Significance levels are indicated as follows: (**) *p* < 0.01, (*) *p* < 0.05. For clarity, non-significant results are not shown.

Nevertheless, given that CTSB, CTSD, MMP-9, and α-B-Cry treatment induced additional fragmentation and loosening of partially degraded “aged” amyloids, we examined potential changes in their effects on HEK-293, HeLa TK-, SK-N-MC, and DF1 cell viability. Analysis revealed no additional reduction in “aged” amyloid toxicity toward various cell lines after treatment with the degradation factors. Conversely, MMP-9, α-B-Cry, and CTSD treatment of “aged” amyloids increased degradation product toxicity for some cell lines (Fig. 4). The absence of reduced cytotoxicity agrees with SDS-PAGE data indicating that the selected cellular factors did not induce additional fibril dissociation into non-toxic monomeric proteins. Furthermore, these findings align with published data showing that incomplete amyloid degradation products often exhibit cytotoxicity comparable to, or exceeding, that of intact amyloids [8, 19, 55]. A logical next step for future work would be to perform a more detailed photophysical and structural analysis of factor-specific degradation products and of the mechanisms underlying their cytotoxicity. In particular, future studies could combine time-resolved LC–MS mapping of released and residual fibrillar fractions with fractionation-preserving structural analysis and membrane-focused assays to define which fibril-derived species are responsible for the preserved or increased cytotoxicity after incomplete amyloid processing.

## Discussion

### Capacity of persisting amyloid fibrils to degrade and lose cytotoxicity

**Fig. 5:**
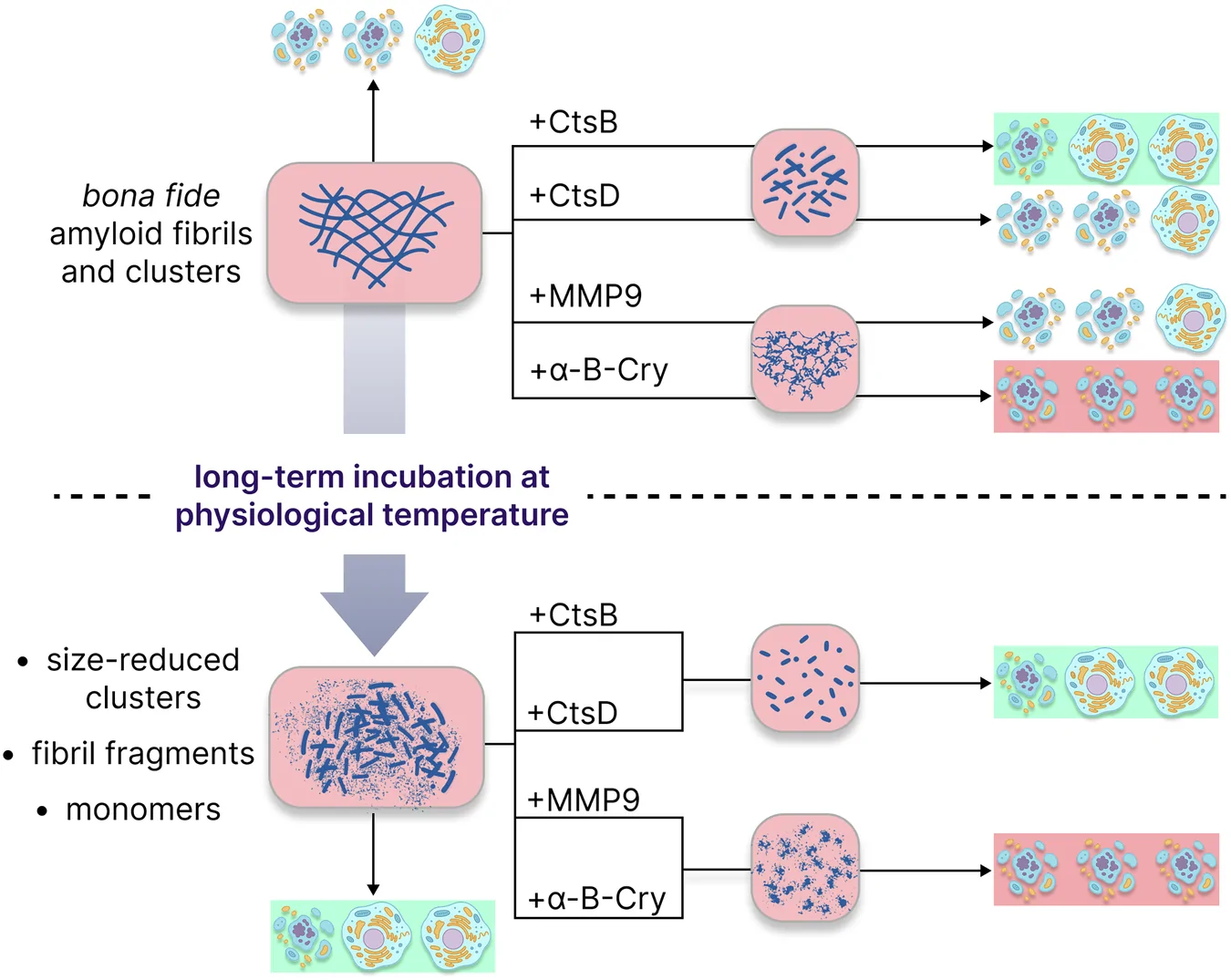
Diagram illustrating the consequences of prolonged incubation of amyloid fibrils at physiological temperature. Observations included over time reductions in amyloid cluster size, separation into individual fibrils, shortening of fibrils, and gradual depolymerization into monomeric subunits, all accompanied by decreased cytotoxicity. Immune-associated proteases, cathepsins B (CTSB), D (CTSD), and matrix metalloproteinase-9 (MMP-9), as well as the chaperone-like protein alpha-B-crystallin (α-B-Cry), more effectively degrade “aged” long-incubated amyloids than “fresh” bona fide amyloid fibrils and their clusters. However, factor-mediated degradation of “aged” amyloids did not reduce cytotoxicity and sometimes increased it. The schematic representation of “fresh” amyloid cytotoxicity after enzyme-induced degradation is based on our previous studies [51–54].

Our study shows that mature lysozyme amyloid fibrils are not structurally inert after assembly. During 16 months of incubation at physiological temperature in a simplified aqueous cell-free model, both fibril polymorphs underwent progressive suprastructural remodeling, including declustering, fibril shortening, monomer accumulation, and attenuation of intrinsic cytotoxicity (Fig. 5 and Table 1). Importantly, the preserved ThT fluorescence lifetime and retained fibrillar morphology of residual aggregates indicate that these changes primarily reflect partial dissociation and suprastructural reorganization rather than complete structural collapse of the remaining cross-β fibrillar population. We refer to this time-dependent post-assembly remodeling as amyloid “aging”.

**Table 1:** Magnitude of changes in “aged”* vs. “fresh”** amyloid fibrils and “aged” intact vs. treated fibrils.

Treatment***	Parameter	Lys2_ Change	Lys6.3_ Change
**None**	β-sheets (%)	↓↓	↓
	α-helices (%)	↑↑↑	-
	β-turns (%)	-	↑↑↑
	“Others” (%)	-	↑↑
	RLS	↓↓↓	↓↓↓
	Ftotal_ThT	↓↓	↓↓↓
	<τ>_ThT	-	-
	aggregated fraction****	↓↓↓	↓
	HeLa TK^−^ viability	↑↑	↑↑
	HEK-293 viability	↑↑	↑↑
	DF1 viability	↑	↑
	SK-N-MC viability	↑	↑
CTSB	HeLa TK^−^ viability	**-**	**-**
	HEK-293 viability	-	-
	DF1 viability	-	-
	SK-N-MC viability	-	-
CTSD	HeLa TK^−^ viability	-	↓
	HEK-293 viability	↓	-
	DF1 viability	↓	-
	SK-N-MC viability	-	-
MMP9	HeLa TK− viability	↓↓	-
	HEK-293 viability	↓↓↓	↓
	DF1 viability	-	-
	SK-N-MC viability	↓	-
α-B-Cry	HeLa TK⁻ viability	↓	↓
	HEK-293 viability	↓	↓
	DF1 viability	-	↓
	SK-N-MC viability	-	↓

^*^“Aged” amyloids: aggregates incubated at physiological temperature for 16 months.

**“Fresh” amyloids: newly prepared aggregates.

***Changes were calculated as (“aged”−“fresh”)/“fresh” × 100% for untreated samples and (treated−intact)/ intact × 100% for treated samples.

In both cases, the change is designated as “↑“ or “↓“ if the obtained value is up to 25%, as «↑↑» or «↓↓» if the value is in the range of 25–50%, as «↑↑↑» or «↓↓↓» if the value exceeds 50%. Upward arrows indicate an increase in the parameter; downward arrows indicate a decrease. Statistically indistinguishable values are indicated by «–».

****The aggregated fraction content was calculated by subtracting the monomeric fraction, quantified using pseudo-native SDS-PAGE and ImageJ processing.

The aging trajectory appeared biphasic, with an active remodeling phase during the first months followed by a plateau after ~10 months. This deceleration may reflect several non-mutually exclusive factors. First, amyloid samples are structurally heterogeneous, and less stable individual fibrils are likely depleted earlier, leaving more resistant clustered or compact fractions. Second, the shorter fragments that accumulate during aging may be mechanically less prone to further breakage than longer, more brittle fibrils, consistent with the strong length-dependence of amyloid fibril mechanics [56–58]. Third, released low-molecular-weight species may remain in dynamic equilibrium with residual fibrillar seeds, limiting complete disassembly. Thus, the plateau does not necessarily indicate the end of molecular exchange, but rather the establishment of a slower remodeling regime dominated by more stable residual assemblies and partially dissociated material.

### Limitations and generalizability of findings

We incubated amyloids in Milli-Q water at physiological temperature. This in vitro system should not be viewed as a direct mimic of the physiological milieu, but rather as a minimalistic long-term aqueous cell-free model designed to resolve the intrinsic features of mature amyloid fibril aging under controlled conditions at physiological temperature. Such a reductionist baseline was important for dissecting the structural and biological consequences of long-term post-assembly remodeling before introducing additional environmental variables. In this simplified system, amyloid aging led to spontaneous partial degradation, altered cytotoxicity, and changed susceptibility to subsequent degrading factors, indicating that fibril age itself can shape the biological outcome of amyloid processing. This baseline framework can now be extended stepwise by incorporating salts, buffering components, macromolecular crowding, extracellular factors, and active clearance mechanisms in order to determine how increasing physiological complexity reshapes the consequences of amyloid aging. Such extension is particularly important because previous studies have shown that environmental variables can substantially alter amyloid behavior, including fibril formation pathways, supramolecular organization, and even fibril-core structure, as demonstrated for HEWL [59], tau [60], and IAPP [61].

An additional limitation is that the released non-fibrillar material was not chemically resolved by LC–MS. Therefore, low-level non-enzymatic modifications, including oxidation, deamidation, or isoaspartate formation, cannot be formally excluded. At the same time, the electrophoretic pattern does not support extensive nonspecific proteolysis: pseudo-native SDS-PAGE showed accumulation of material in the monomeric lysozyme migration region without a sub-monomeric fragment ladder, while the residual fibrillar fraction retained a stable ThT fluorescence lifetime, consistent with preservation of the local ThT-binding microenvironment. If subtle non-enzymatic modifications contribute to long-term fibril remodeling, they should be considered a potential mechanistic component of post-assembly amyloid aging. This possibility is biologically plausible, because slow chemical remodeling is a known feature of long-lived proteins in vivo, including protein oxidation [62] and deamidation [63], and related isoaspartate/pyroglutamate or oxidative modifications have been documented for amyloidogenic peptides such as Aβ during aging and Alzheimer’s disease [64–66].

We also performed our studies only for a limited set of systems, precluding definitive conclusions about the universality of these effects. However, published data indicate that amyloid degradation mechanisms typically depend not on the identity of the amyloid-forming protein but rather on the degradation trigger (e.g., amyloid-degrading factors, environmental conditions) [19]. We therefore propose that the aging phenomena observed here likely extend to other amyloid systems. We selected lysozyme fibril polymorphs as model systems, enabling us to represent different clustering degrees and morphologies that influence protein aggregate structural stability and cytotoxicity. “Fresh” Lys2 amyloids consisted predominantly of individual fibrils and loosely packed clots, whereas “fresh” Lys6.3 samples assembled predominantly into large, dense clusters. Such variation in clustering degree and supramolecular organization is a common feature of amyloid polymorphism across different protein systems. For instance, β2-microglobulin fibrils, whose accumulation causes dialysis-related amyloidosis [67], display polymorphism and clot-forming propensity that depends on incubation conditions and protein sequence length, influencing aggregate cytotoxicity [68–70]. Similarly, β-amyloid peptide fibrils accumulating in Alzheimer’s disease [71] form polymorphic strains with different clustering degrees even under identical conditions [72]. For IAPP fibrils found in the pancreas of individuals with type 2 diabetes [73, 74], the repertoire of amyloid polymorphs changes with protein sequence length [75]. Finally, α-synuclein amyloids involved in Parkinson’s disease pathogenesis [76, 77] exhibit different aggregate polymorphs ranging from individual fibrils to massive clusters, each with distinct physicochemical properties, cytotoxicity, and susceptibility to perturbations [78–80].

Despite these limitations, our results provide a foundation for understanding potential reorganization and degradation mechanisms of amyloid aggregates with varying clustering degrees over time. The shared cross-β architecture and similar polymorphic behavior across diverse amyloidogenic proteins [1, 81] suggest that the aging phenomena documented here may represent a general feature of amyloid aggregate dynamics. Validation in other amyloid systems will be necessary to confirm the broader applicability of these patterns.

### Efficient degradation of “aged” amyloids fails to mitigate cytotoxicity and can even exacerbate it

Our analysis of amyloid degradation by lysosomal proteases involved in the immune response, as well as a small heat shock protein α-B-Cry, revealed that the modes of degradation are conserved regardless of fibril maturity. CTSB and CTSD induced fragmentation, whereas MMP-9 and α-B-Cry induced “loosening” and destructuring of both “fresh” and “aged” lysozyme amyloids (Fig. 5). Notably, individual thin fibrils and their fragments, which accumulate during aggregate aging, underwent more efficient degradation by external factors compared to freshly prepared amyloids. This enhanced susceptibility of aged aggregates to proteolytic and chaperone-mediated processing represents one of the key findings of our study. Although protease and α-B-Cry treatment of “aged” amyloids generated more loosened, smaller, and less clustered fibrils than treatment of “fresh” amyloids, we observed no reduced cytotoxicity of their degradation products compared to intact “aged” amyloids (Table 1).

This persistence or increase in cytotoxicity is most conservatively interpreted as evidence that incomplete processing of aged fibrils generates biologically active fibril-derived material rather than fully detoxified monomeric protein. This interpretation is particularly relevant for MMP-9 and α-B-Cry, because their dominant morphological effect is not complete fibril depolymerization, but rather loosening, decompaction, and partial disorganization of the external fibrillar architecture. This conclusion is consistent with our previous studies on freshly prepared amyloid fibrils, where MMP-9 and α-B-Cry induced analogous fibril loosening/decompaction rather than complete monomerization, and where such remodeling was associated with preserved or increased cytotoxicity depending on the amyloid system and cell type [52, 54]. More broadly, our previous factor-specific studies showed that CTSB, CTSD, MMP-9, and α-B-Cry remodel mature amyloid fibrils through distinct structural routes, including fragmentation/destruction for cathepsins [51, 53] and loosening/decompaction for MMP-9 and α-B-Cry [52, 54].

We did not directly isolate the toxic entities or measure their membrane activity in the present study. Nevertheless, the proposed model is supported by several converging observations. First, TEM shows that MMP-9- and α-B-Cry-treated aged samples contain loosened and partially disassembled fibrillar material rather than unchanged intact fibrils or fully monomerized protein. Second, pseudo-native SDS-PAGE indicates that these treatments do not produce a corresponding increase in monomeric lysozyme, arguing against complete detoxifying depolymerization. Third, independent studies support a direct link between the external organization of amyloid fibrils and their biological activity: the surface structure of Aβ fibrils contributes to cytotoxicity [82], amyloid fibrils can perturb membrane organization and promote abnormal stabilization of membrane raft domains [83], and the dynamic fuzzy coat of amyloid fibrils has been proposed to participate in cell-membrane binding and surface-mediated interactions [84]. Thus, the increased toxicity observed after MMP-9- and α-B-Cry-mediated processing may arise from increased exposure, mobility, or reorganization of peripheral fibrillar elements rather than from complete disassembly into soluble oligomers.

The modest cytotoxicity increases observed after CTSD treatment of aged lysozyme fibrils may reflect a related but mechanistically distinct consequence of fragmentation. Unlike MMP-9- and α-B-Cry-mediated loosening, fragmentation increases the number of fibril ends and generates shorter fibrillar species, which can display enhanced biological activity. This is consistent with previous evidence that fibril fragmentation enhances amyloid cytotoxicity [85, 86] and that short α-synuclein fibrils are more neurotoxic than long fibrils because their higher end density promotes the release of toxic oligomeric species [87]. Thus, different modes of incomplete amyloid processing may preserve or increase cytotoxicity through distinct structural routes: external decompaction and surface reorganization in the case of MMP-9/α-B-Cry, and fibril shortening/end enrichment in the case of fragmentation-dominated remodeling. While the precise toxic species remain to be chemically and biophysically defined, the available data support the conclusion that incomplete processing of aged fibrils can preserve or enhance biological activity by altering the accessible external architecture or size distribution of fibrillar assemblies. The size-dependent cytotoxicity of amyloid assemblies, with intermediate-sized fragments often exhibiting greater toxicity than either intact fibrils or fully dissociated monomers, represents an important consideration for understanding the biological consequences of aggregate reorganization [85, 88, 89].

More broadly, the biological consequences of amyloid remodeling during fibril persistence are unlikely to be determined by a single parameter alone [8]. Rather, they appear to depend on a complex interplay between the prior structural state of the fibrils and the mechanism by which a given factor perturbs the amyloid assembly. Different amyloid-interacting molecules can act at distinct stages of the aggregation/remodeling landscape, including primary nucleation, secondary nucleation, elongation, fibril-surface catalysis, or local fibril destabilization. Accordingly, they may generate structurally different released species and, therefore, different toxic outcomes even when acting on the same amyloid system. This principle is illustrated by BRICHOS, whose anti-amyloid activity depends on assembly state [90] and, in Aβ systems, can selectively suppress secondary nucleation [91], and by the recently described bacterial protein CagA, which was reported to inhibit amyloid formation across multiple substrates and stages of fibrillization [92]. In this context, the present results identify fibril age as an additional variable likely to shape the outcome of amyloid processing.

### Implications for understanding amyloid aggregate dynamics and future directions

Prolonged amyloid persistence is a defining feature of many amyloid-associated pathologies, but the in vivo environment is more complex than the reductionist system used here. Cellular components, macromolecular crowding, enzymatic degradation, immune responses, ongoing monomer supply, extracellular cofactors, post-translational modifications, and local physicochemical conditions can all influence fibril stability and remodeling [8, 19, 93–96]. Nevertheless, the present model isolates an intrinsic property of mature amyloid assemblies: their biological behavior can change as a function of post-assembly residence time. In vivo, such remodeling could have opposing consequences. Partial dissociation may reduce direct cytotoxic burden, whereas accumulation of short fibrillar fragments could increase seeding capacity, tissue dissemination, or membrane-associated toxicity [8, 85, 86, 88, 89]. Thus, amyloid aging may attenuate some toxic properties while simultaneously creating structurally remodeled species with distinct pathogenic potential.

The present study was designed around mature fibrils as a model of persistent amyloid assemblies and is therefore most directly relevant to pathological or pathology-associated amyloid deposits. To better define the boundaries of this relevance, it is useful to consider functional amyloids, which represent a biologically distinct class of amyloid assemblies. Functional amyloids are typically formed, maintained, and disassembled under tight spatial and temporal regulation and are linked to physiological roles rather than uncontrolled accumulation [97]. This is illustrated by Pmel in pigmentation [98], the RIP1/RIP3 necrosome in signaling [99], and hnRNPDL-2 fibrils as a stable and non-toxic functional amyloid [100]. Accordingly, direct extrapolation of our long-term model of spontaneous amyloid aging to functional amyloids would be premature. Nevertheless, the more general principle that amyloid maturity may influence structural stability and biological outcome could be worth testing in regulated functional amyloid systems in future work.

The differential susceptibility of fresh and aged fibrils to degradation factors also has therapeutic implications. Current amyloid-imaging modalities, including PET [101], amyloid-specific magnetic resonance imaging (MRI) [102], and fluorescence/optoacoustic imaging [103], detect amyloid deposits but do not resolve their post-assembly age or remodeling state. Future approaches capable of distinguishing amyloid populations by probe binding, antibody accessibility, structural organization, or degradation susceptibility could help evaluate whether a given deposit is likely to resist clearance, undergo fragmentation, or generate biologically active degradation products.

Collectively, our findings provide the first in vitro evidence that mature amyloid aggregates undergo spontaneous time-dependent reorganization, resulting in suprastructural transition and attenuated cytotoxicity (effectively “age”). Importantly, we demonstrate that this reorganization fundamentally alters aggregate susceptibility to proteolytic and chaperone-mediated processing, with “aged” fibrils being degraded more efficiently yet sometimes yielding more cytotoxic products than intact aggregates. These results establish aggregate maturity as a critical parameter governing both the intrinsic properties and the biological fate of amyloid assemblies. Our findings have potential implications for understanding the consequences of prolonged amyloid persistence in vivo. Future research should examine how cellular factors influence amyloid degradation dynamics, the molecular mechanisms generating cytotoxic degradation products in vivo, and develop multifactorial therapeutic approaches accounting for amyloid aggregate “age”. This proves particularly relevant given ongoing discussions of modern anti-amyloid immunotherapies, where aggressive plaque degradation can trigger amyloid-related imaging abnormalities (ARIA), including brain edema (ARIA-E), microhemorrhage (ARIA-H) [104, 105], refractory status epilepticus, and fatal cerebral arteritis [106, 107]. Approaches that incorporate not only the amyloid-forming protein type and deposit localization but also “temporal characteristics” of amyloid assemblies may improve our understanding of resistance to therapeutic interventions and advance personalized medicine.

In conclusion, our data support a view of mature amyloid deposits not as static end-products of protein misfolding, but as dynamic entities whose “age” shapes their biological interactions. By demonstrating that spontaneous remodeling governs both intrinsic pathogenicity and susceptibility to biological clearance, we identify fibril maturity as a critical variable in amyloid biology. Crucially, the paradoxical observation that the facilitated disassembly of “aged” fibrils by cellular factors fails to eliminate or even exacerbates cytotoxicity suggests that effective therapies must account for this temporal dimension to ensure that accelerating amyloid clearance does not inadvertently trigger cell death.

## Materials and methods

### Materials

Thioflavin T (ThT) from AnaSpec (USA), buffer components, lysozyme from chicken egg white, and 3-(4,5-dimethylthiazol-2-yl)-2,5-diphenyltetrazolium bromide (MTT) from Sigma (USA) were used as received without further purification. Cell culture reagents were obtained from Gibco (Thermo Fisher Scientific, USA). Culture flasks and flat-bottom 96-well plates were purchased from Corning (USA). CTSD from human liver was obtained from Sigma (USA). CTSB was acquired from Merck (Germany). Eukaryotic MMP-9 was purchased from CLOUD-CLONE CORP (USA). α-B-crystallin isolation and purification were performed as described previously [108–110] with some modifications (see Supplemental material).

### Sample preparation

Recombinant hen egg-white lysozyme amyloid aggregates were prepared in vitro at a concentration of 2 mg/mL using two protocols involving incubation for 2 days under intense stirring: 1) in 20% acetic acid in the presence of 100 mM NaCl, pH 2.0, at 37 °C (Lys2); 2) in 50 mM KH2PO4-NaOH buffer in the presence of 3 M GdnHCl, pH 6.3, at 57 °C (Lys6.3). After fibrillogenesis, mature amyloid fibrils were dialyzed against Milli-Q water. For the long-term aging experiments, post-dialysis fibril suspensions were stored in sealed vials, in darkness, at 37 °C, with pH monitoring throughout the 16-month incubation period. Prior to amyloid degradation experiments, fibrils were resuspended in conditions favorable for protease and α-B-crystallin activity. Strictly acidic pHs were avoided to prevent the potential effect of charge repulsion between amyloids and ThT dye [111], thereby excluding false positive results in the amyloid degradation assay. Amyloid-degrading factors were activated and deactivated using previously established protocols [112–116] (see Supplemental material).

### Transmission electron microscopy

Amyloid fibrils were visualized using a Libra 120 transmission electron microscope (Carl Zeiss, Germany). Samples (10 μL) were applied onto formvar/carbon-coated copper grids for 1.5 min. Excess liquid was removed, the grids were negatively stained with 1% aqueous uranyl acetate for 1.5 min, air-dried, and imaged.

### Confocal microscopy

ThT-stained amyloid aggregates were examined using a Leica TCS SP5 confocal laser scanning microscope (Leica Microsystems GmbH, Germany). Imaging was conducted with a 60x oil immersion objective lens (numerical aperture 1.4) under 405 nm laser excitation. Images compared within the same experiment were acquired using identical microscope settings. Quantitative analysis of amyloid aggregate size distribution was carried out in Fiji (NIH, Bethesda, MD, USA). Confocal images were converted to 8-bit grayscale, binarized using the thresholding algorithm, and the areas occupied by fluorescent aggregates in the resulting binary images were measured. The same image-processing workflow was applied to all analyzed images.

### Spectral measurements

A U-3900H spectrophotometer (Hitachi, Japan) was utilized to record the absorption spectra of the samples. To account for light scattering, the absorption spectra of amyloid fibrils and their mixtures with ThT were corrected following a standard protocol [51, 117].

Fluorescence measurements were conducted using a Cary Eclipse spectrofluorimeter (Varian, Australia), with ThT fluorescence excited at 440 nm. All fluorescence intensity data were corrected for the primary inner filter effect [118]. RLS was recorded at 530 nm (*λ*_ex_ = 530 nm).

CD spectra were acquired using a J-810 spectropolarimeter (Jasco, Japan). Far-UV CD spectra were collected over the 190–260 nm range with 0.2 nm increments and standard sensitivity, employing a 1 mm path length cuvette. To optimize the signal-to-noise ratio and minimize light scattering, sample concentrations were kept relatively low (0.15–0.2 mg/mL). The absence of distortion of the recorded amyloid spectra due to light scattering was confirmed by near-zero CD values in the 250–260 nm region (where positive values are usually observed with high light scattering of samples). Spectra were scanned at 10 nm/min with an 8-second response time to further enhance data quality. Three scans were averaged and baseline-corrected using Milli-Q water as a reference. The secondary structure content of fibrils was estimated using their corrected far-UV CD spectra by the BeStSel web server (https://bestsel.elte.hu) [119, 120]. BeStSel method is considered suitable for estimating the secondary-structure content of amyloid aggregates and β-structure-rich proteins.

Fluorescence decay curves of ThT bound to amyloid fibrils were collected using a spectrometer FluoTime 300 (PicoQuant, Germany) with the Laser Diode Head LDH-C-440 (*λ*_ex_ = 440 nm) at *λ*_em_ = 490 nm. The decay curves were analyzed in FluoFit (PicoQuant). The decay curves were fitted by numerical reconvolution of the model function with the experimentally measured instrument response function (IRF), using nonlinear least-squares minimization based on the Levenberg–Marquardt algorithm. The IRF was measured experimentally before each sample using scattered light from a dilute Ludox colloidal silica solution and was explicitly included in the reconvolution fit. Multi-exponential models were used, and the minimal number of components required to achieve reduced *χ*² values close to 1 and randomly distributed residuals was selected. In practice, two- or three-exponential models were usually sufficient. The intensity-weighted mean fluorescence lifetime of bound ThT was used as the reported lifetime parameter.

### Equilibrium microdialysis

Solutions of ThT with amyloid fibrils were prepared through equilibrium microdialysis using a Harvard Apparatus/Amika (USA) system [121, 122] (see Supplemental material).

### MTT assay

Cervical epithelioid carcinoma (HeLa TK-), human embryonic kidney (HEK-293), neuroepithelioma (SK-N-MC), and dermal fibroblasts (DF1) cell lines from the shared research facility “Vertebrate Cell Culture Collection” were utilized in the study. All cell lines were authenticated by STR profiling and verified to be free of mycoplasma contamination. Metabolic activity was assessed by the MTT assay 24 h after treatment with the tested amyloids at a final concentration of 0.003 mg/mL, following a previously established standard protocol [51, 123, 124].

### Denatured and pseudo-native sodium dodecyl sulfate (SDS) gel electrophoresis

Denatured SDS-PAGE was carried out following a standard protocol [125, 126]. Prior to loading, samples were mixed with Laemmli SDS-PAGE sample buffer containing 2% SDS (Bio-Rad, Hercules, CA, USA) and boiled for 5 minutes. For pseudo-native conditions, samples were prepared in a modified buffer (0.0625 M Tris-HCl, pH 6.8, 1% SDS, 10% glycerol, and 0.002% bromophenol blue) and loaded onto the gel without boiling. For pseudo-native SDS-PAGE quantification, the full migration region corresponding to monomeric lysozyme ( ~ 14.5 kDa) was integrated in ImageJ and normalized to the corresponding monomer control lane within the same gel subpanel. This approach was used because intact disulfide-rich proteins analyzed under nonreducing/nonboiled SDS conditions may show slight band broadening/blurring or non-ideal electrophoretic mobility due to differences in retained compactness and SDS binding [127–129].

### Long-term storage and sterility controls

For long-term aging experiments, post-dialysis amyloid fibrils were stored in sealed vials, protected from light, at 37 °C. The pH of the samples was monitored throughout the incubation period and remained stable. Gross sample evaporation was controlled procedurally by monitoring the liquid level in sealed vials using external level marks. No visible decrease in sample volume was detected during storage. Protein concentration was additionally monitored in representative samples by absorbance at 280 nm after appropriate dilution and scattering correction, and no increase consistent with evaporation-driven concentration was observed. As an end-point sterility control, aliquots (10 µL) of representative fresh and 16-month-aged Lys2 and Lys6.3 samples were plated on antibiotic-free LB agar and incubated at 37 °C for 72 h. Plate visualization was performed after incubation at 37 °C for 24, 48, and 72 h. The *E. coli* BL21(DE3) strain was used as a positive control.

### Statistical analysis

All quantitative data are presented as mean ± standard deviation (SD) from five independent measurements (*n* = 5) for “fresh” and “aged” amyloids. Statistical analyses were performed using GraphPad Prism version 10 (GraphPad Software, San Diego, California, USA). For time-course data, statistical comparisons were performed separately for Lys2 and Lys6.3 fibrils using Brown–Forsythe and Welch ANOVA tests, followed by Dunnett’s T3 multiple comparisons test with each time point compared to the corresponding 0-month sample. For pairwise comparisons, Welch’s *t* test was used. Significance levels are indicated as follows: *p* < 0.01 (**), *p* < 0.05 (*), and *p* ≥ 0.05 (ns). For clarity, non-significant results are not shown in the figures unless stated otherwise.

## Supplementary information

Supplemental material

## Data Availability

The data that support the findings of this study are available from the corresponding author upon reasonable request.
