# Supplementary material for: Spontaneous aging of mature amyloids alters structural stability, cytotoxicity, and susceptibility to biological clearance
Source: Cell Death Discov. 2026 Jul 7;12:390. doi: 10.1038/s41420-026-03245-1 (PMC13627696; doi:10.1038/s41420-026-03245-1)
Supplement: Supplementary file 1 [file 41420_2026_3245_MOESM1_ESM.docx]

**Spontaneous aging of mature amyloids alters structural stability, cytotoxicity, and susceptibility to biological clearance**

**Maksim I. Sulatsky^1,†^, Olga V. Stepanenko^2,†^, Arina A. Kayda^2^, Olesya V. Stepanenko^2^, Ekaterina V. Mikhailova^2^, Anna I. Sulatskaya^2,^***

^1^ Laboratory of cell morphology, Institute of Cytology Russian Academy of Sciences, 4 Tikhoretsky ave., 194064 St. Petersburg, Russia; [m_sulatsky@mail.ru](mailto:m_sulatsky@mail.ru)

^2^Laboratory of structural dynamics, stability and folding of proteins, Institute of Cytology Russian Academy of Sciences, 4 Tikhoretsky ave., 194064 St. Petersburg, Russia; [arina.nurova@gmail.com](mailto:arina.nurova@gmail.com), sov@incras.ru; lvs@incras.ru; 4evamkh@gmail.com; ansul@mail.ru

^†^These authors contributed equally to the work

*****Corresponding author: Anna I. Sulatskaya, Laboratory of structural dynamics, stability and folding of proteins, Institute of Cytology Russian Academy of Sciences, 4 Tikhoretsky ave., 194064 St. Petersburg, ansul@mail.ru

**Details of experimental procedures**

**Protein production and purification**

The α-B-crystallin was synthesized in BL21(*DE3*) host cells (Invitrogen, USA) using pET28a vector. Bacterial cells were grown in LB medium supplemented with kanamycin. The expression of α-B-crystallin was initiated by 0.5 mM IPTG at 28 °C overnight. α-B-crystallin isolation and purification was performed as described previously ^1, 2^ with some modifications. The cell pellet was subjected to a freeze-thaw cycle. After sonication, DNA was degraded by the treatment with DNase I at a final concentration of 2.5 µg/L and the suspension was for 45 min at 37 °C. The crude cell lysate was fractionated by ammonium sulfate (the target protein precipitates by 40% ammonium sulfate). The subsequent steps of the α-B-crystallin purification included ion-exchange chromatography on Mono Q 5/50GL (GE Healthcare, Sweden) and size-exclusion chromatography on Superose 12 10/300GL (GE Healthcare, Sweden). The protein was highly concentrated (up to 6-10 mg/ml) and stored in 25 mM TrisHCl, 0.1 mM EDTA, 50 mM NaCl, 0.1 mM PMSF, 1 mM DTT, pH 7.6. The purity of the protein was tested by SDS/PAGE in 12% polyacrylamide gels ^3^. The protein concentration was calculated using an extinction coefficient at 280 nm of 14 100 M^−1^cm^−1^ according to amino acids composition.

**Protease’s activation**

CTSD was self-activated in 20 mM sodium-acetate buffer pH 4.0, 1 mM EDTA ^4^, by incubation at 37 °C for 30 minutes ^5, 6^. Pre-activated CTSD was then added to the amyloid fibrils at a weight ratio of 1/50. Strictly acidic pHs were avoided to prevent the potential effect of charge repulsion between amyloids and ThT dye ^7^, thereby excluding false positive results in the amyloid degradation assay. Pre-activated in 50 mM sodium acetate buffer (pH 5)/1 mM EDTA with 1 mM dithiothreitol (DTT) according to the manufacturing protocol, CTSB was added to the amyloid fibrils (without or with crowders) in a 1/25 weight ratio. Since CTSD and CTSB are not thermostable ^6, 8^, short-term heating at 60 °C was used to terminate their activity. MMP-9 was activated in TNCB buffer (50mM TrisHCl (pH7.4), 50mM NaCl, 5mM CaCl_2_, 0.05%Brij35) with trypsin at a 4:1 weight ratio for 8 h at 37 °C ^9^, after which 1mM phenylmethylsulfonyl fluoride (PMSF) was applied to inactivate trypsin. Pre-activated in this way MMP-9 was added to amyloid at a weight ratio of 1/25. 1 mM EDTA was used to inactivate MMP-9 ^9^. The freshly thawed α-B-crystallin was added at a molar ratio of 0.5:1 to the fibril suspensions dissolved in 20 mM NaH2PO4/Na2HPO4 (pH 7.4) ^10^.

**Equilibrium microdialysis**

Solutions of ThT with amyloid fibrils were prepared through equilibrium microdialysis using a Harvard Apparatus/Amika (USA) system ^11, 12^. The procedure was carried out with fibril concentrations of 0.5 mg/ml and ThT at 30 µM. The amount of free ThT was determined by measuring absorbance at 412 nm, applying a molar extinction coefficient of 31,600 M⁻¹cm⁻¹ ^13^.

**Figure S1.** Sterility control of lysozyme amyloid samples. Representative "fresh" and "aged" Lys2 and Lys6.3 samples were plated on LB agar and incubated at 37 °C for 72 h. No colony growth was detected in any tested sample, arguing against viable microbial contamination during long-term storage. *E.coli* BL21(DE3) strain was used as a positive control (control).

**Figure S2.** Secondary structure content in the samples with lysozyme amyloid fibrils incubated at physiological temperature. α-Helices (left column), β-turns (middle column), and "others" component, which includes 3₁₀-helices, π-helices, β-bridges, bends, loop/irregular, and structurally unresolved regions (right column) content calculated by BeStSel server are presented. Data for Lys2 and Lys6.3 are presented in the top and bottom row, respectively. Data are presented as mean ± SD, n=5 at each time point. Statistical comparisons were performed separately for each condition using Brown–Forsythe and Welch ANOVA tests, followed by Dunnett’s T3 multiple comparisons test with each time point compared to the corresponding 0-month sample. Significance levels are indicated as follows: (**) p < 0.01, (*) p < 0.05; ns, not significant. For clarity, non-significant results are not shown.

**Figure S3.** The stability of amyloid fibrils after buffer exchange. Visualization by transmission electron microscopy. Microphotographs of amyloids prepared in acidic pH (Lys2, top rows) and in near-neutral pH (Lys6.3, bottom rows) fibrils after long-term incubation in Milli-Q water and resuspending in the buffer solutions favoring CTSB, CTSD, MMP9, and α-B-crystallin (α-B-Cry) activity are shown. Scale bars are equal to 1 µm.

**Figure S4.** Pseudo-native 17 % SDS-PAGE of the fresh and aged Lys2 amyloid fibrils after exposure to CTSB, CTSD, MMP9, and α-B-crystallin. Samples of "fresh" and "aged" Lys2 amyloids before (fresh, aged) and after (fresh+CTSB, aged+CTSB, fresh+CTSD, aged+CTSD, fresh+MMP9, aged+MMP9, fresh+α-B-Cry, aged+α-B-Cry) treatment were loaded in lanes 1-4. As controls, monomeric lysozyme (mono, lane 5) was loaded at a concentration equal to that of amyloids, and α-B-crystallin (α-B-Cry, lane 6) was loaded at the concentration corresponding to that in the amyloid samples. Marker proteins (M) were loaded in the last lanes; molecular weights are shown on the right.

**References:**

1.	Datskevich PN, Mymrikov EV, Sluchanko NN, Shemetov AA, Sudnitsyna MV, Gusev NB. Expression, purification and some properties of fluorescent chimeras of human small heat shock proteins. *Protein expression and purification* 2012, **82**(1)**:** 45-54.

2.	Mymrikov EV, Bukach OV, Seit-Nebi AS, Gusev NB. The pivotal role of the beta 7 strand in the intersubunit contacts of different human small heat shock proteins. *Cell stress & chaperones* 2010, **15**(4)**:** 365-377.

3.	Laemmli UK. Cleavage of structural proteins during the assembly of the head of bacteriophage T4. *Nature* 1970, **227**(5259)**:** 680-685.

4.	Benes P, Vetvicka V, Fusek M. Cathepsin D—Many functions of one aspartic protease. *Critical Reviews in Oncology/Hematology* 2008, **68**(1)**:** 12-28.

5.	Hasilik A, von Figura K, Conzelmann E, Nehrkorn H, Sandhoff K. Lysosomal enzyme precursors in human fibroblasts. Activation of cathepsin D precursor in vitro and activity of beta-hexosaminidase A precursor towards ganglioside GM2. *Eur J Biochem* 1982, **125**(2)**:** 317-321.

6.	Conner GE, Richo G. Isolation and characterization of a stable activation intermediate of the lysosomal aspartyl protease cathepsin D. *Biochemistry* 1992, **31**(4)**:** 1142-1147.

7.	Sulatsky MI, Stepanenko OV, Stepanenko OV, Kuznetsova IM, Turoverov KK, Sulatskaya AI. Prediction of the Feasibility of Using the <<Gold Standard>> Thioflavin T to Detect Amyloid Fibril in Acidic Media. *Analytical chemistry* 2024, **96**(5)**:** 2158-2164.

8.	Gounaris AD, Slater EE. Cathepsin B from human renal cortex. *Biochem J* 1982, **205**(2)**:** 295-302.

9.	Okada Y, Gonoji Y, Naka K, Tomita K, Nakanishi I, Iwata K*, et al.* Matrix metalloproteinase 9 (92-kDa gelatinase/type IV collagenase) from HT 1080 human fibrosarcoma cells. Purification and activation of the precursor and enzymic properties. *J Biol Chem* 1992, **267**(30)**:** 21712-21719.

10.	Stepanenko OV, Sulatsky MI, Mikhailova EV, Stepanenko OV, Povarova OI, Kuznetsova IM*, et al.* Alpha-B-Crystallin Effect on Mature Amyloid Fibrils: Different Degradation Mechanisms and Changes in Cytotoxicity. *International Journal of Molecular Sciences* 2020, **21**(20).

11.	Kuznetsova IM, Sulatskaya AI, Uversky VN, Turoverov KK. A new trend in the experimental methodology for the analysis of the thioflavin T binding to amyloid fibrils. *Mol Neurobiol* 2012, **45**(3)**:** 488-498.

12.	Kuznetsova IM, Sulatskaya AI, Uversky VN, Turoverov KK. Analyzing thioflavin T binding to amyloid fibrils by an equilibrium microdialysis-based technique. *PLoS One* 2012, **7**(2)**:** e30724.

13.	Sulatskaya AI, Lavysh AV, Maskevich AA, Kuznetsova IM, Turoverov KK. Thioflavin T fluoresces as excimer in highly concentrated aqueous solutions and as monomer being incorporated in amyloid fibrils. *Sci Rep* 2017, **7**(1)**:** 2146.
